# Frameshift mutations of *YPEL3* alter the sensory circuit function in *Drosophila*

**DOI:** 10.1242/dmm.042390

**Published:** 2020-06-03

**Authors:** Jung Hwan Kim, Monika Singh, Geng Pan, Adrian Lopez, Nicholas Zito, Benjamin Bosse, Bing Ye

**Affiliations:** 1 Department of Biology, University of Nevada, Reno, Reno, NV 89557, USA; 2 Life Sciences Institute and Department of Cell and Developmental Biology, University of Michigan, Ann Arbor, MI 48109, USA

**Keywords:** YPEL3, Pathogenicity, Rare mutation, Synaptic connection

## Abstract

A frameshift mutation in *Yippee-like* (*YPEL*) *3* was recently found from a rare human disorder with peripheral neurological conditions including hypotonia and areflexia. The YPEL gene family is highly conserved from yeast to human, but its members’ functions are poorly defined. Moreover, the pathogenicity of the human *YPEL3* variant is completely unknown. We generated a *Drosophila* model of human *YPEL3* variant and a genetic null allele of *Drosophila* homolog of *YPEL3* (referred to as *dYPEL3*). Gene-trap analysis suggests that *dYPEL3* is predominantly expressed in subsets of neurons, including larval nociceptors. Analysis of chemical nociception induced by allyl-isothiocyanate (AITC), a natural chemical stimulant, revealed reduced nociceptive responses in both *dYPEL3* frameshift and null mutants. Subsequent circuit analysis showed reduced activation of second-order neurons (SONs) in the pathway without affecting nociceptor activation upon AITC treatment. Although the gross axonal and dendritic development of nociceptors was unaffected, the synaptic contact between nociceptors and SONs was decreased by the *dYPEL3* mutations. Furthermore, expressing dYPEL3 in larval nociceptors rescued the behavioral deficit in *dYPEL3* frameshift mutants, suggesting a presynaptic origin of the deficit. Together, these findings suggest that the frameshift mutation results in *YPEL3* loss of function and may cause neurological conditions by weakening synaptic connections through presynaptic mechanisms.

## Abstract (precis)

**Summary:** A *Drosophila* model of a human *YPEL3* variant was generated and used to discover that the human variant alters neuronal communication through a loss-of-function mechanism.

## INTRODUCTION

*YPEL3* belongs to the Yippee-like gene family, which is composed of a number of genes in eukaryotic species ranging from yeast to human (Hosono et al., 2004). Only a handful of studies have hinted at the biological roles of YPEL3. YPEL3 was initially identified as a small unstable apoptotic protein because of its low protein stability and the ability to induce apoptosis when overexpressed in a myeloid cell line (Baker, 2003). Subsequent studies implicated YPEL3 as a tumor suppressor. YPEL3 expression correlates with p53 activity (Kelley et al., 2010). Overexpression and knockdown analyses suggest that YPEL3 suppresses the epithelial-to-mesenchymal transition in cancer cell lines by increasing GSK3β expression (Zhang et al., 2016). Other studies have shown the role of YPEL genes in development. The loss-of-function mutations of YPEL orthologs in ascomycete fungus altered fungal conidiation and appressoria development (Han et al., 2018). In zebrafish, a morpholino-mediated targeting of *ypel3* altered brain structures (Blaker-Lee et al., 2012).

Recently, a mutation in human *YPEL3* was found in a patient with a rare disorder that manifests a number of neurological symptoms [the National Institutes of Health (NIH)-Undiagnosed Diseases Program]. The mutation was caused by duplication of a nucleotide in a coding exon of *YPEL3*, resulting in a frameshift and consequently a premature stop codon. The clinical observation showed that the patient had normal cognition but manifested peripheral symptoms, including areflexia and hypotonia. However, whether the identified *YPEL3* mutation is pathogenic in the nervous system is unknown. Moreover, little is known about the functions of YPEL3 in the nervous system.

In the present study, we generated a *Drosophila* model of the human condition by creating the disease-relevant *YPEL3* variant using CRISPR/Cas9-mediated in-del mutations. Our gene-trap analysis suggests that subsets of neurons, including nociceptors, express the *Drosophila* homolog of *YPEL3* (referred to as *dYPEL3*). Subsequent analysis revealed reduced nociceptive behavior in *dYPEL3* mutants. Consistently, we found that *dYPEL3* mutations impaired the activation of second-order neurons (SONs) in the nociceptive pathway and reduced the synaptic contact between nociceptors and these SONs. We further demonstrate that the behavioral, circuit and cellular phenotypes in the *dYPEL3* frameshift mutants are recapitulated in a genetically null allele of *dYPEL3*, and that expressing wild-type dYPEL3 in nociceptors rescues the altered nociceptive behavior in the frameshift mutants. These findings suggest that the identified human *YPEL3* mutation is pathogenic and affects neuronal synapses through a loss-of-function mechanism.

## RESULTS

### Generation of a disease-relevant variant of *YPEL3* in *Drosophila*

**Fig. 1.**
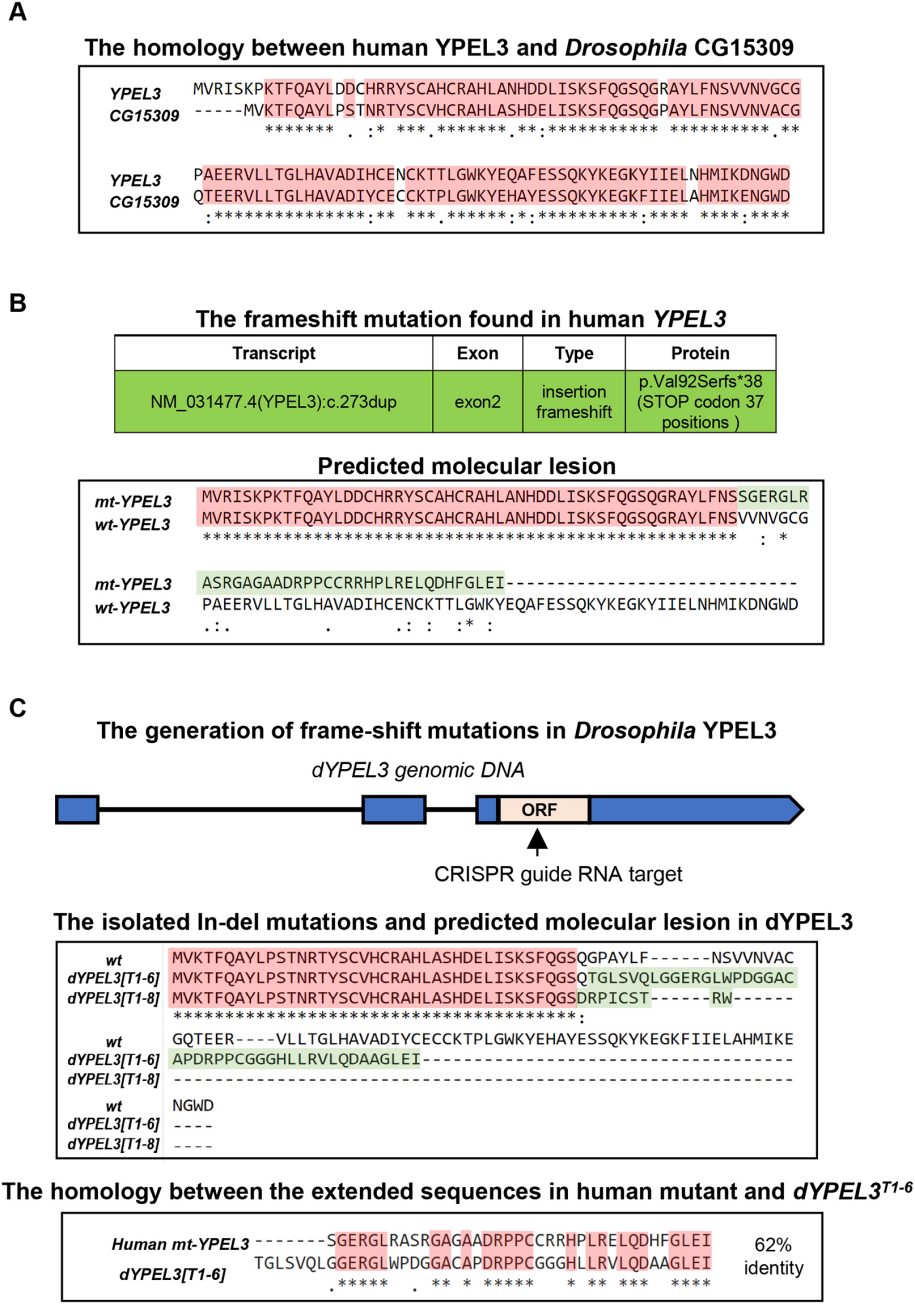
**The generation of a *Drosophila* model of *YPEL3* frameshift mutation.** (A) *CG15309* is the *Drosophila* homolog of human *YPEL3*. Sequence alignment between human YPEL3 (YPEL3) and *Drosophila* CG15309. Shaded in pink are the identical amino acid sequences. (B) Duplication of a cytosine nucleotide in *YPEL3* gene from a patient (top). A predicted molecular lesion in human *YPEL3* (bottom) introduces an ectopic amino acid sequence (shaded in green). The preserved region is shaded in pink. (C) CRISPR-Cas9 mediated in-del mutation in *CG15309/dYPEL3*. A guide RNA is designed targeting the middle of the coding exon (top). The isolated *dYPEL3* in-del mutants (middle). Sequence alignment between wild type (*wt*), *dYPEL3^T1-6^* and *dYPEL3^T1-8^*. The introduced ectopic amino acid sequences following a premature stop codon are shaded in green. The sequence alignment of the introduced ectopic amino acid sequences from the human *YPEL3* frameshift mutants and *dYPEL3^T1-6^* (bottom). The identical amino acid sequences are shaded in pink. ORF, open reading frame.

Although the discovery of a *YPEL3* variant in a patient underscores the importance of YPEL3 in human health, whether this variant causes any deficits in the nervous system is unknown. There are five YPEL genes in human: *YPEL1*-*YPEL5*. YPEL1, YPEL2, YPEL3 and YPEL4 are highly homologous to each other (up to 96% identity at amino acid sequences), whereas YPEL5 has only ∼40% homology to the other members (Hosono et al., 2004). We found two YPEL homologs in *Drosophila*, *Yippee* and *CG15309*, using an ortholog search (Hu et al., 2011). The predicted amino acid sequences of CG15309 showed 88% similarity (81% identity) to human YPEL3 (Fig. 1A), while that of Yippee showed 65% similarity (53% identity) (data not shown). *Yippee* appears to be an ortholog of *YPEL5* because it is more closely related to YPEL5 than YPEL3, with 87% similarity and 73% identity to YPEL5 (data not shown). Therefore, we named *CG15309* as *dYPEL3*.

The variant identified in the human patient introduces an extra nucleotide in the middle of the coding exon, which produces a frameshift and consequently results in the incorporation of the 37 ectopic amino acids followed by a premature stop codon (Fig. 1B). To generate a *Drosophila* model of the human variant, we took advantage of the CRISPR/Cas9 technology to induce in-del mutations (Port et al., 2014). The entire coding sequence of *dYPEL3* is in a single exon. We designed a guide RNA that targets the middle of the coding exon (Fig. 1C, top) and successfully isolated two *dYPEL3* frameshift mutants named *dYPEL3^T1-6^* and *dYPEL3^T1-8^* (Fig. 1C, middle). *dYPEL3^T1-6^* has a two-nucleotide deletion at 121 nucleotides downstream of a start codon, which generated a premature stop codon at 153 nucleotides downstream of a start codon, while *dYPEL3^T1-8^* carries a four-nucleotide deletion at 118 nucleotides downstream of a start codon, and generated a premature stop codon at 145 nucleotides downstream of a start codon. Similar to the human variant, the mutations introduced additional amino acids followed by a premature stop codon (Fig. 1C, middle). The ectopic amino acids in *dYPEL3^T1-6^* closely resemble those of the human variant (Fig. 1C, bottom).

### *dYPEL3* is expressed in subsets of neurons

**Fig. 2.**
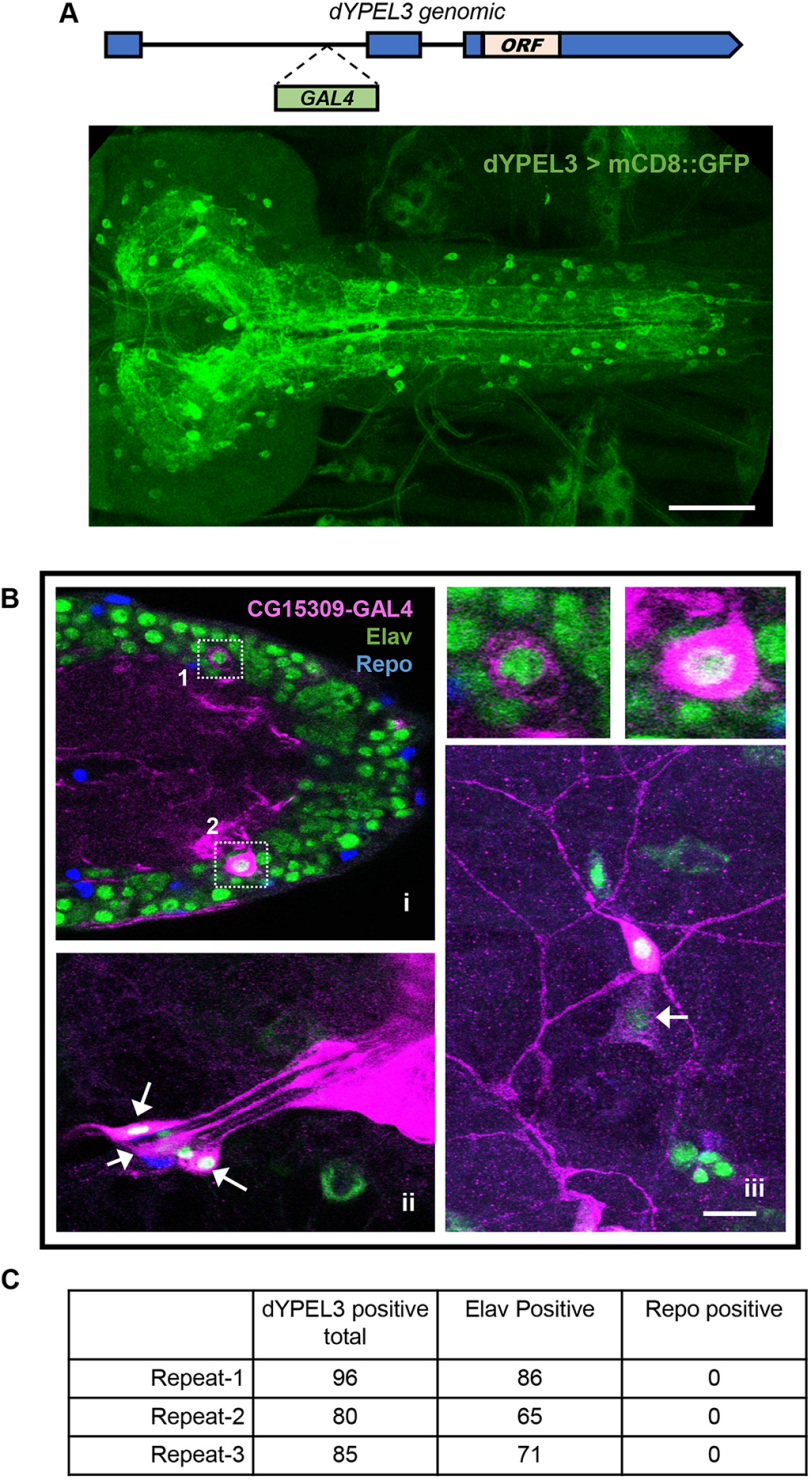
***dYPEL3* is a neuronal gene.** (A) The expression pattern of dYPEL3 in the CNS. The InSITE gene trap line for *dYPEL3* was used (*CG15309-GAL4/dYPEL3-GAL4*). GAL4 transcription factor is inserted in the first intron. The introduction of *UAS-mCD8::GFP* demonstrates the endogenous expression pattern of *dYPEL3*. Note that the *CG15309-GAL*-positive cells elaborate fine processes throughout the CNS. Scale bar: 50 µm. (B) mCD8::GFP (magenta) was expressed under *dYPEL3-GAL4* following immunostaining with anti-Elav (neuronal, green) and anti-Repo (glial, blue) antibodies. (i) The CNS. Cell bodies in 1 and 2 are shown magnified on the top right. (ii,iii) Chordotonal neurons (ii, arrows) and a class III da neuron (iii) in the PNS. iii also shows a class IV da neuron (nociceptor) that is positive for dYPEL3 (arrow). Scale bar: 10 µm. (C) Quantitation of the Elav-positive and Repo-positive cells that are labeled with *CG15309-GAL4*. The majority of dYPEL3-postive cells were Elav positive, but none were positive for Repo.

We did not find any gross developmental defects in *dYPEL3^T1-6^* or *dYPEL3^T1-8^* flies. Homozygotes were viable and fertile, and showed normal growth under standard culture condition (data not shown). This raises the possibility that dYPEL3 is expressed in a subset of cells in the body. Our efforts of generating antibodies against dYPEL3 failed in two independent trials, precluding the use of immunostaining for identifying the cell types that express dYPEL3. We thus took advantage of a GAL4 enhancer-trap line, *CG15309-GAL4* (*dYPEL3-GAL4*) (Gohl et al., 2011), to study the expression pattern of dYPEL3 in flies. This line contains a GAL4 insertion in the first intron of *dYPEL3*, which places the GAL4 under the control of the endogenous *dYPEL3* promoter and enhancers (Fig. 2A, top). We expressed a membrane GFP reporter (mouse CD8::GFP or mCD8::GFP) to visualize *dYPEL3* expression pattern in *Drosophila* larvae. A small number of cells in the larval central nervous system (CNS), including the ventral nerve cord and brain, were labeled by mCD8::GFP (Fig. 2A, bottom). These cells extended fine processes that cover most of the neuropil area in the larval CNS, suggesting that they are neurons. To identify the cell types that express dYPEL3, *dYPEL3-GAL4>mCD8::GFP* samples were co-immunostained with the neuron marker anti-Elav and the glial marker anti-Repo (Fig. 2B). Approximately 85% of cells that were labeled with *dYPEL3-GAL4* were positive for Elav, but none were positive for Repo (Fig. 2C). This result suggests that dYPEL3 is predominantly expressed in neurons, but not in glia. Interestingly, *dYPEL3-GAL4* also labeled a subset of sensory neurons, including the class IV dendritic arborization (da) neurons (nociceptors), class III da neurons and chordotonal neurons (both mechanosensors), but not the class I da neurons (proprioceptors) (Fig. 2Bii,iii; Fig. S1). dYPEL3 was not expressed in muscles or epidermal cells (Fig. S2).

### The disease-relevant mutations of *dYPEL3* reduce nociceptive behavioral responses

**Fig. 3.**
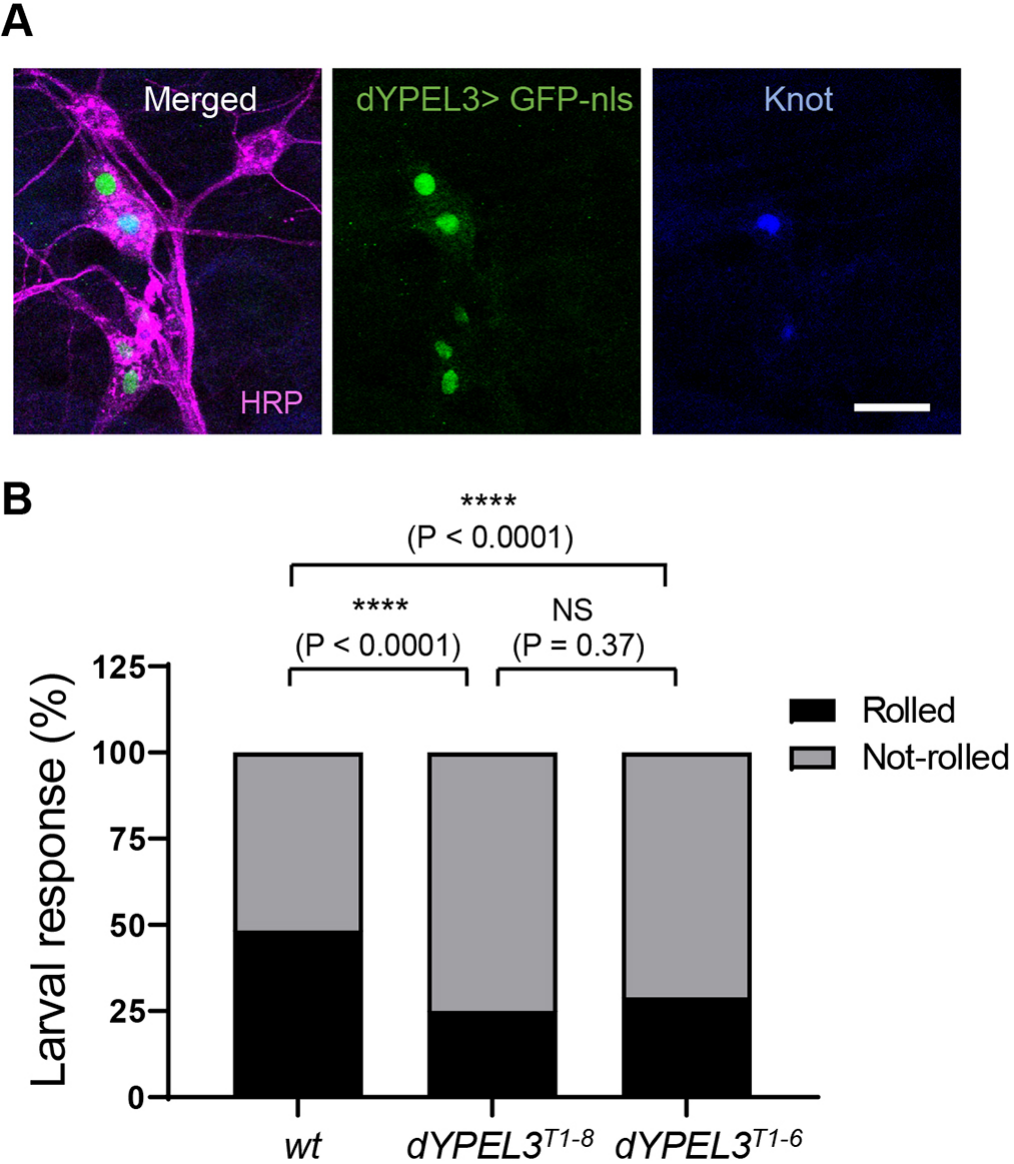
***dYPEL3* frameshift mutations reduce nociceptive behavior.** (A) Nociceptive/class IV da neurons are positive for dYPEL3. A nuclear GFP (GFP-nls, green) was expressed under *dYPEL3-GAL4* following immunostaining with anti-Knot antibody (blue). Anti-HRP antibody was used to label all PNS neurons (magenta). Scale bar: 10 μm. (B) The AITC-induced nociceptive behavior was measured in a wild-type control (*wt*) and *dYPEL3* frameshift mutants (*dYPEL3^T1-8^* and *dYPEL3^T1-6^*). The number of larvae that exhibited complete rolling behavior was scored and expressed as a percentage (*n*=252 for each genotype). The Chi-squared test was performed between the groups. NS, non-significant; ****P<0.0001.

The human patient shows symptoms mainly in the peripheral nervous system (PNS), including areflexia and hypotonia (the NIH-Undiagnosed Diseases Program). We thus focused our analysis on dYPEL3-positive neurons in the PNS (Fig. 2Bii,iii). The enhancer-trap analysis suggests that nociceptors express dYPEL3. This finding was confirmed by co-immunostaining with the nociceptor marker anti-Knot antibody (Hattori et al., 2007; Jinushi-Nakao et al., 2007) (Fig. 3A). The nociceptors detect various stimuli, including noxious heat, touch and chemicals, and activate the nociceptive pathway that leads to the nocifensive rolling behavior (Hwang et al., 2007). Allyl-isothiocyanate (AITC), a natural chemical stimulant, has been proposed to cause larval nociceptive behavior through nociceptors (Kaneko et al., 2017; Xiang et al., 2010; Zhong et al., 2012). Since AITC also elicits nociceptive behavior in adult flies through gustatory sensory neurons (Soldano et al., 2016), we determined whether AITC-induced nocifensive rolling required the larval nociceptors. Optogenetic inhibition of larval nociceptors with *Guillardia theta* anion channel rhodopsin-1 (GtACR1) (Mohammad et al., 2017) dramatically reduced AITC-induced rolling (Fig. S3), demonstrating that the AITC-induced rolling depends on the nociceptors on the larval body wall.

We first determined whether the functions of nociceptors were altered by the *dYPEL3* mutations. We applied AITC to the wild-type control, *dYPEL3^T1-6^* and *dYPEL3^T1-8^* and found a significant reduction in nociceptive rolling behavior in the *dYPEL3* mutants (48% and 40% reduction, respectively, Fig. 3B). The extent of decrease in nociceptive rolling was not different between the two mutant alleles of *dYPEL3*, which are almost identical except for the sequences in the ectopic stretch of amino acids (Fig. 1C). This suggests that the truncation of dYPEL3, but not the presence of the ectopic amino acid sequences, is responsible for the observed phenotype. *dYPEL3^T1-8^* represents a simpler version since it only has incorporation of a few ectopic amino acids (Fig. 1C). Therefore, we focused our analysis on *dYPEL3^T1-8^* for further analysis.

**Fig. 4.**
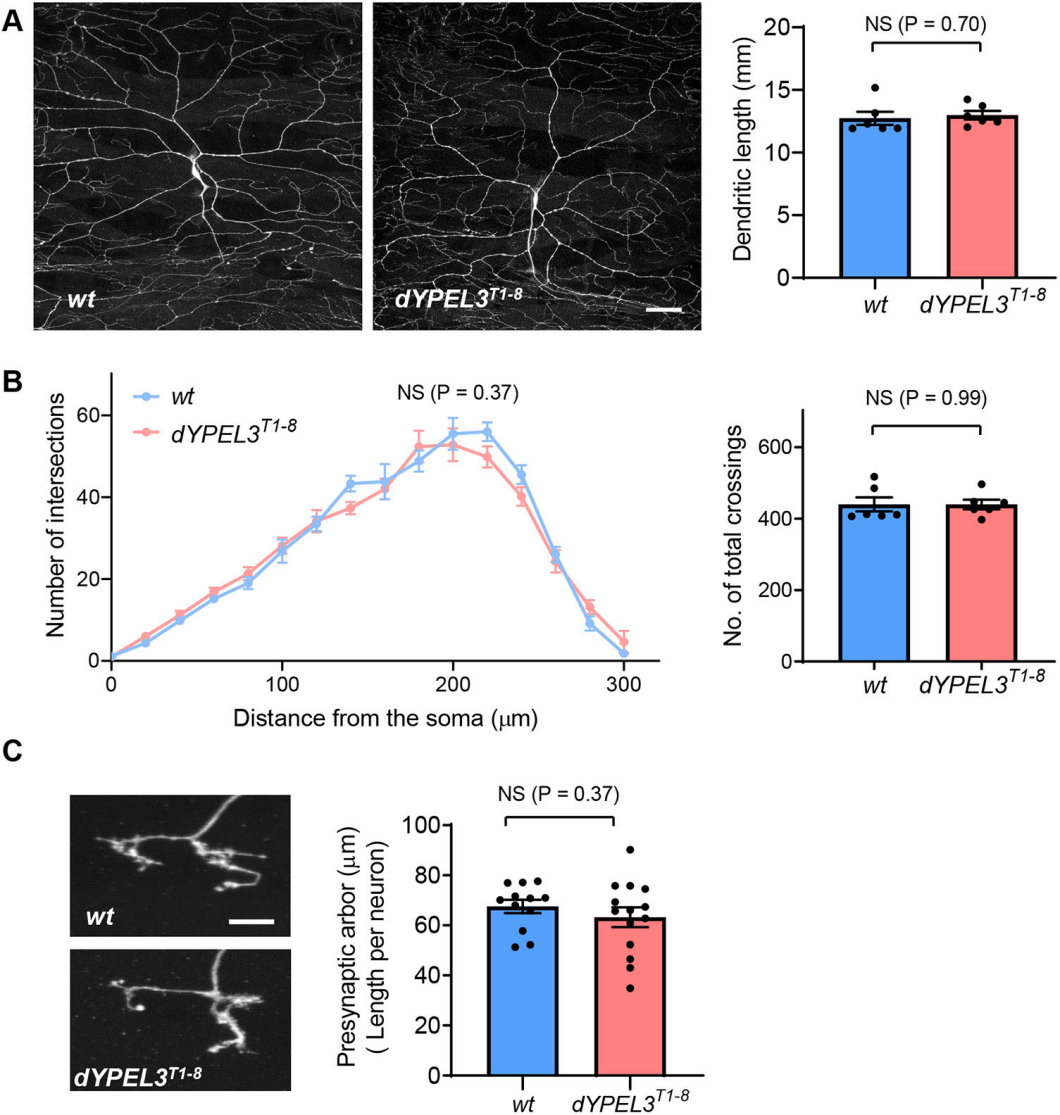
**The development of nociceptors is not altered by *dYPEL3* frameshift mutations.** (A) mCD8::GFP was specifically expressed in nociceptors using *ppk-GAL4* in wild-type control (*wt*) and *dYPEL3* frameshift mutants (*dYPEL3^T1-8^*). Total length of dendrites was measured (*n*=6 for each genotype). Unpaired Student’s *t*-test with Welch's correction was performed. Scale bar: 50 µm. (B) Sholl analysis was performed with 20-µm radius increment from the dendritic tracing. Number of total crossings within 300 µm from the cell center was measured. Two-way ANOVA for Sholl analysis and unpaired Student's *t*-test with Welch's correction for total dendritic crossings were performed. (C) The axon terminals of single nociceptors from wild type and *dYPEL3^T1-8^* mutants were visualized using the flip-out technique. The total length of axon terminals was measured (*n*=12 for *wt*, *n*=14 for *dYPEL3^T1-8^*). Scale bar: 10 µm. Unpaired Student's *t*-test with Welch's correction was performed. Data are presented as mean±s.e.m. All statistical analysis was two-tailed. NS, non-significant.

How does *dYPEL3* mutation affect the sensory function? We first looked into whether the *dYPEL3* mutation affects the development of nociceptors. We expressed mCD8::GFP specifically in nociceptors in wild-type and *dYPEL3^T1-8^* larvae using the nociceptor-specific driver *ppk-GAL4* (Grueber et al., 2007). The dendritic arborization was assessed using Sholl analysis (Sholl, 1953) and by measuring total dendritic length. *dYPEL3^T1-8^* mutations did not alter the dendritic development (Fig. 4A,B). Next, we tested whether the presynaptic terminals of nociceptors are defective in *dYPEL3* mutants. To this end, a flip-out mosaic experiment was performed to label single nociceptive presynaptic arbors (Yang et al., 2014). The total length of the presynaptic arbor of each nociceptor was indistinguishable between wild type and *dYPEL3^T1-8^* (Fig. 4B), suggesting that *dYPEL3^T1-8^* does not affect the development of presynaptic arbors.

### The disease-relevant mutations of *dYPEL3* reduce the synaptic transmission from nociceptors to their postsynaptic neurons

**Fig. 5.**
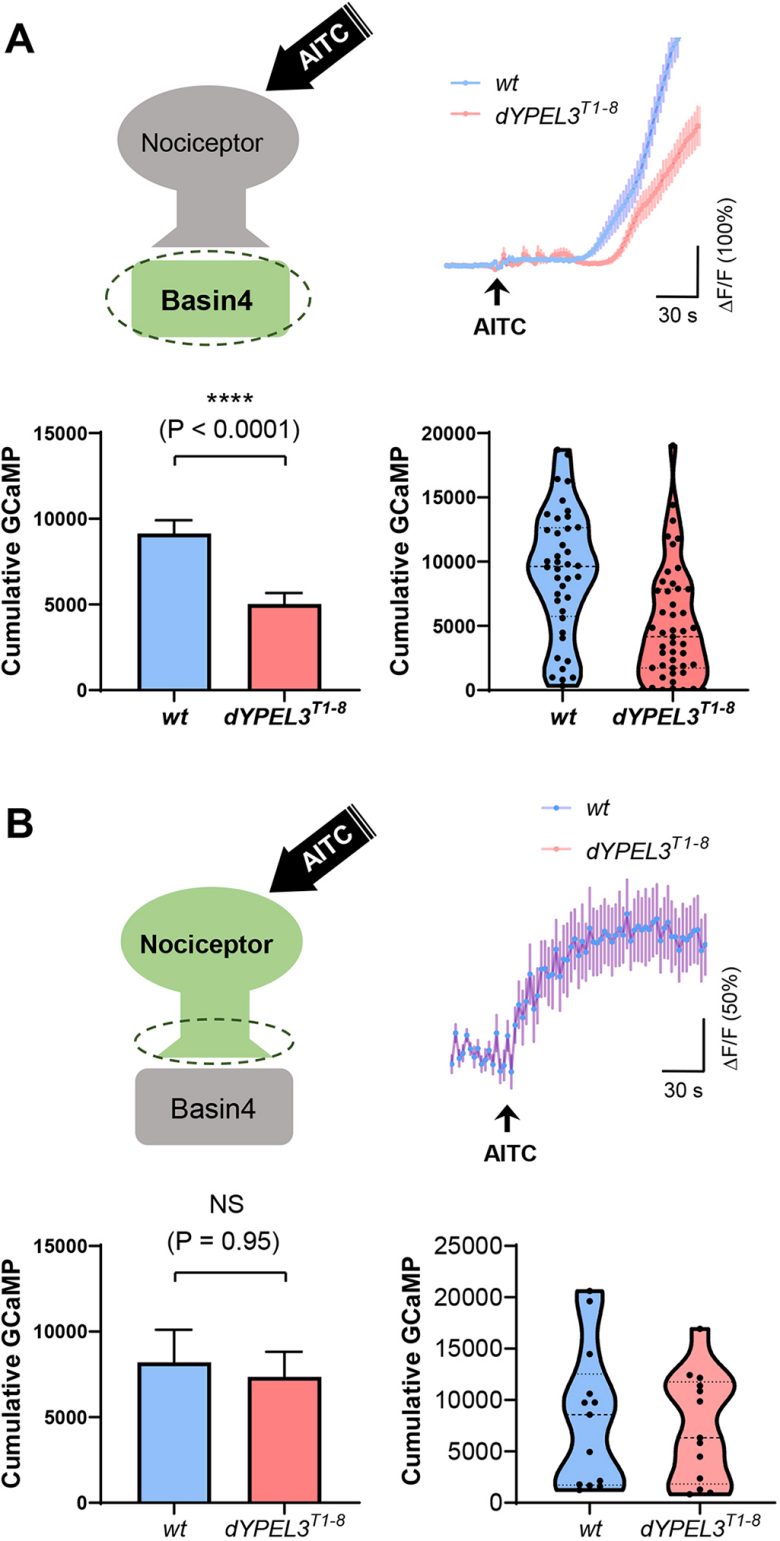
***dYPEL3* frameshift mutations reduce the synaptic transmission from nociceptors to Basin-4 neurons.** (A) Basin-4 activation upon AITC treatment was reduced by *dYPEL3^T1-8^*. GCaMP6f was expressed in Basin-4 neurons. Nociceptors were activated with 10 mM AITC (top left). Ca^2+^ increase in Basin-4 was measured by GCaMP fluorescence and the tracing over time is shown (*n*=40 for *wt*, *n*=47 for *dYPEL3^T1-8^*) (top right). The cumulative GCaMP activation from single Basin-4 neurons was measured and presented as mean±s.e.m. (bottom left), as well as in a violin plot to show distribution (bottom right). Mann–Whitney test. (B) Nociceptor activation was not altered by *dYPEL3* mutations. GCaMP6f was expressed in nociceptors using *ppk-GAL4*. Nociceptors were activated with 10 mM AITC (top left). Ca^2+^ increase in the axon terminals of nociceptors was measured by GCaMP fluorescence and the tracing over time is shown (*n*=13 for each genotype) (top right). The cumulative GCaMP activation from the nociceptor axon terminals was measured and presented as mean±s.e.m. (bottom left), as well as in a violin plot to show distribution (bottom right). Mann–Whitney test. All statistical analysis was two-tailed. NS, non-significant; ****P<0.0001.

Next, we assessed the synaptic transmission from nociceptors to their postsynaptic neuron Basin-4, a key SON in the nociceptive pathway (Ohyama et al., 2015). The activation of Basin-4 elicits nociceptive behavior even in the absence of nociceptor activation, while silencing these neurons suppresses nociceptive behavior (Ohyama et al., 2015). The genetically encoded calcium indicator GCaMP6f was selectively expressed in Basin-4 for recording intracellular calcium, a proxy of neuronal activity (Chen et al., 2013) (Fig. 5A). Larvae were dissected in insect saline as a fillet preparation with intact PNS and CNS (Kaneko et al., 2017) and treated with AITC to stimulate the nociceptors. AITC elicited robust GCaMP signals that persisted over several minutes in both nociceptors and Basin-4 neurons (Fig. 5A,B). We found that the GCaMP signals in Basin-4 neurons were significantly decreased in *dYPEL3^T1-8^* mutants, compared to wild-type control (Fig. 5A, 43% decrease). By contrast, GCaMP measurement in nociceptor axon terminals showed that *dYPEL3^T1-8^* did not change AITC-induced activation of nociceptors (Fig. 5B).

### The disease-relevant mutations of *dYPEL3* reduce the synaptic contact between nociceptors and their postsynaptic neurons

How do the *dYPEL3* mutations reduce the nociceptor-to-Basin-4 synaptic transmission? To address this, we employed a synaptic-contact-specific GFP reconstitution across synaptic partners (GRASP) technique, termed syb-GRASP (Macpherson et al., 2015), to assess the synaptic contact between the presynaptic terminals of nociceptors and the dendrites of Basin-4 neurons. The GRASP technique utilizes two separate fragments of GFP molecule – split-GFP1-10 (spGFP1-10) and split-GFP11 (spGFP11), which can be detected by a specific anti-GFP antibody only when the two fragments are in close proximity to reconstitute a complete GFP. In syb-GRASP, spGFP1-10 is fused to the synaptic vesicle protein Synaptobrevin and expressed in the presynaptic neurons, whereas spGFP11 is fused to a general membrane tag and expressed in postsynaptic neurons. Two independent binary gene expression systems, *GAL4-UAS* and *LexA-LexAop*, were used to drive the expression of spGFP1-10 and spGFP11 in different cell types (del Valle Rodríguez et al., 2012). Synaptic vesicle exocytosis from presynaptic terminals exposes spGFP1-10 onto the presynaptic cleft, where it reconstitutes the functional GFP molecule by associating with postsynaptic spGFP11 molecules. This technique has been used widely to visualize synaptic contact between two identified neuron types.

**Fig. 6.**
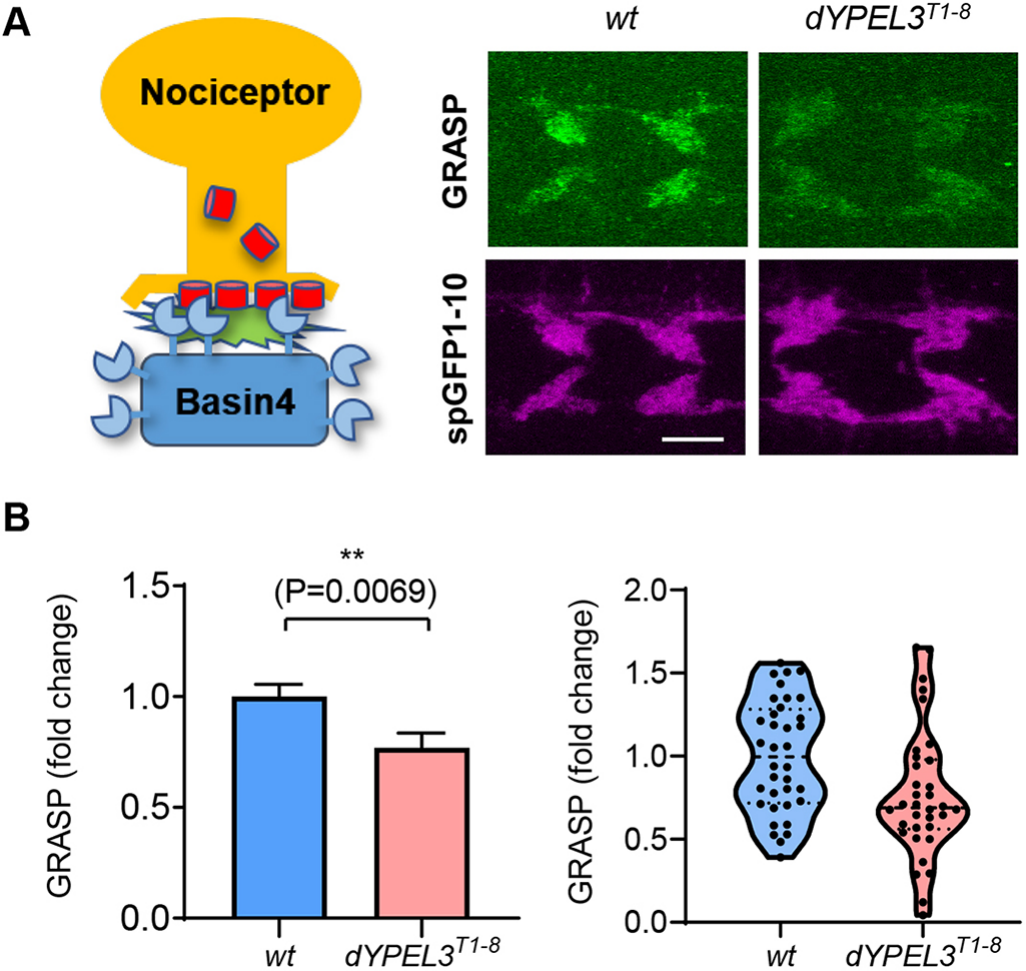
***dYPEL3* frameshift mutations reduce the synaptic contact between nociceptors and Basin-4 neurons.** (A) The syb-GRASP technique was used to report the synaptic contact between nociceptors and Basin-4. The spGFP1-10 (red cylinders) and spGFP11 (blue sectors) were expressed in nociceptors and Basin-4, respectively (left). The resulting GRASP signal was visualized by anti-GRASP antibody (green), and the spGFP1-10 that is expressed in nociceptor axon terminals was used as a normalization control (magenta) (right). Scale bar: 10 μm. (B) The GRASP intensity from each neuropil was normalized by spGFP1-10 intensity and presented as mean±s.e.m. (left), as well as in a violin plot to show distribution (right) (*n*=36 for wt, *n*=34 for *dYPEL3^T1-8^*). Mann–Whitney test. All statistical analysis was two-tailed. **P<0.01.

The spGFP1-10 and spGFP11 were specifically expressed in nociceptors and Basin-4 neurons, respectively (Fig. 6A, left). The resulting GRASP signal was measured in each segmental neuropil, and normalized by the spGFP1-10 intensity in wild type and in *dYPEL3^T1-8^* (Fig. 6A, right). We detected a mild, but significant, decrease (23%) in the GRASP signals in *dYPEL3^T1-8^*, compared to those in wild-type control (Fig. 6B). This suggests that the synaptic contact between nociceptors and its synaptic target Basin-4 is compromised in *dYPEL3^T1-8^*.

### The disease-relevant frameshift mutant of *dYPEL3* is a loss-of-function allele

**Fig. 7.**
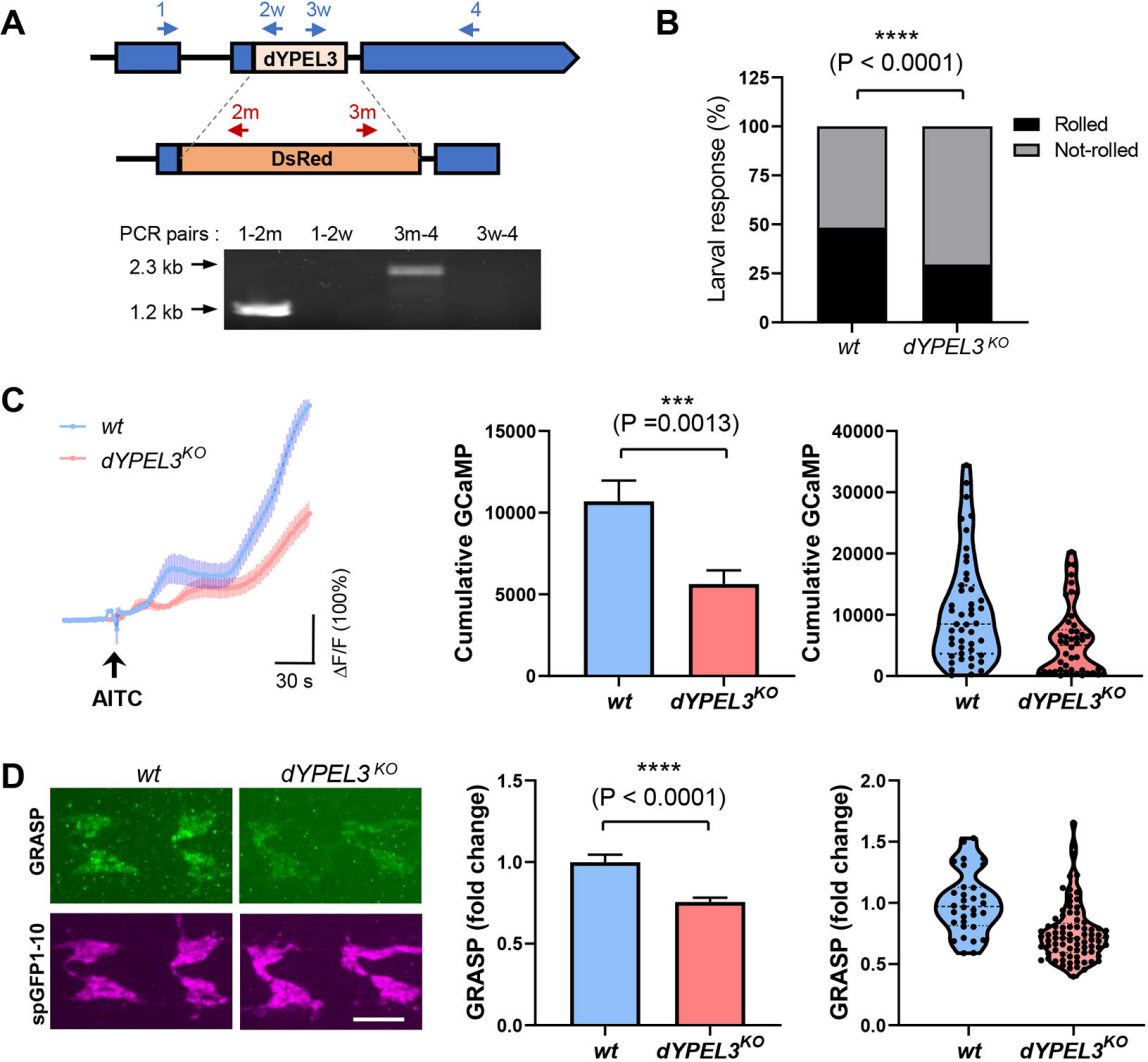
***dYPEL3* knockout mutants recapitulate the phenotypes in the *dYPEL3* frameshift mutants.** (A) The generation of dYPEL3 knockout (*dYPEL3^KO^*) flies. Top: the entire *dYPEL3* ORF was deleted using CRISPR/Cas9-mediated homology-directed recombination. Bottom: agarose gel image of PCR-based genotyping. Note that *dYPEL3^KO^* showed the PCR amplifications specific for DsRed-cassette, but not the ones from wild type. (B) *dYPEL3* knockout reduces AITC-induced behavioral responses. AITC-induced nociceptive behavior was measured in a wild-type control (*wt*) and *dYPEL3* knockout mutants (*dYPEL3^KO^*). The number of larvae that exhibited complete rolling behavior was scored and expressed as a percentage (*n*=252 for *wt*, *n*=203 for *dYPEL3^KO^*). The Chi-squared test was performed between the groups. (C) *dYPEL3* knockout caused a reduction in Basin-4 activation upon AITC. GCaMP6f was expressed in Basin-4 neurons. Nociceptors were activated with 10 mM AITC. Ca^2+^ increase in Basin-4 was measured by GCaMP fluorescence and the GCaMP trace over time is shown (*n*=47 for *wt*, *n*=46 for *dYPEL3^KO^*) (left). The cumulative GCaMP activation from single Basin-4 neurons was measured and presented as mean±s.e.m. (middle), as well as in a violin plot to show distribution (right). Mann–Whitney test. (D) *dYPEL3* knockout causes a reduction in syb-GRASP signals between nociceptors and Basin-4. Synaptobrevin-spGFP1-10 and spGFP11 were expressed in nociceptors and Basin-4, respectively. The resulting GRASP signal was visualized by anti-GFP antibody that only recognizes the reconstituted GFP (anti-GRASP) (green), and the spGFP1-10 expressed in nociceptor axon terminals was used as a normalization control (magenta) (left). Scale bar: 10 μm. The GRASP intensity from each neuropil was normalized by spGFP1-10 intensity and presented as mean±s.e.m. (middle), as well as in a violin plot to show distribution (right) (*n*=32 for wt, *n*=80 for *dYPEL3^T1-8^*). Mann–Whitney test. ***P<0.001 and ****P<0.0001.

The mutations in the patient and in our *Drosophila* model introduce premature stop codons, which may induce the nonsense-mediated decay (Chang et al., 2007), resulting in *YPEL3* loss of function. However, the frameshift mutation in *YPEL3* may escape from nonsense-mediated decay because the premature stop codons are in the last coding exons (both in human and *Drosophila*), which may lead to the production of a truncated version of YPEL3 proteins. To discern these possibilities, we generated a genetically null allele of *dYPEL3* (*dYPEL3^KO^*) by removing the entire *dYPEL3* coding region using CRISPR/Cas9 (Fig. 7A). We found that *dYPEL3^KO^* larvae recapitulated all the deficits found in *dYPEL3* frameshift mutants to the similar extent. These include AITC-induced rolling behavior (40% decrease), AITC-induced Basin-4 activation (47% decrease), and synaptic contact between nociceptors and Basin-4 (24% decrease) (Fig. 7B-D). These results strongly suggest that the disease-relevant mutation of *dYPEL3*, *dYPEL3^T1-8^*, is a loss-of-function allele.

**Fig. 8.**
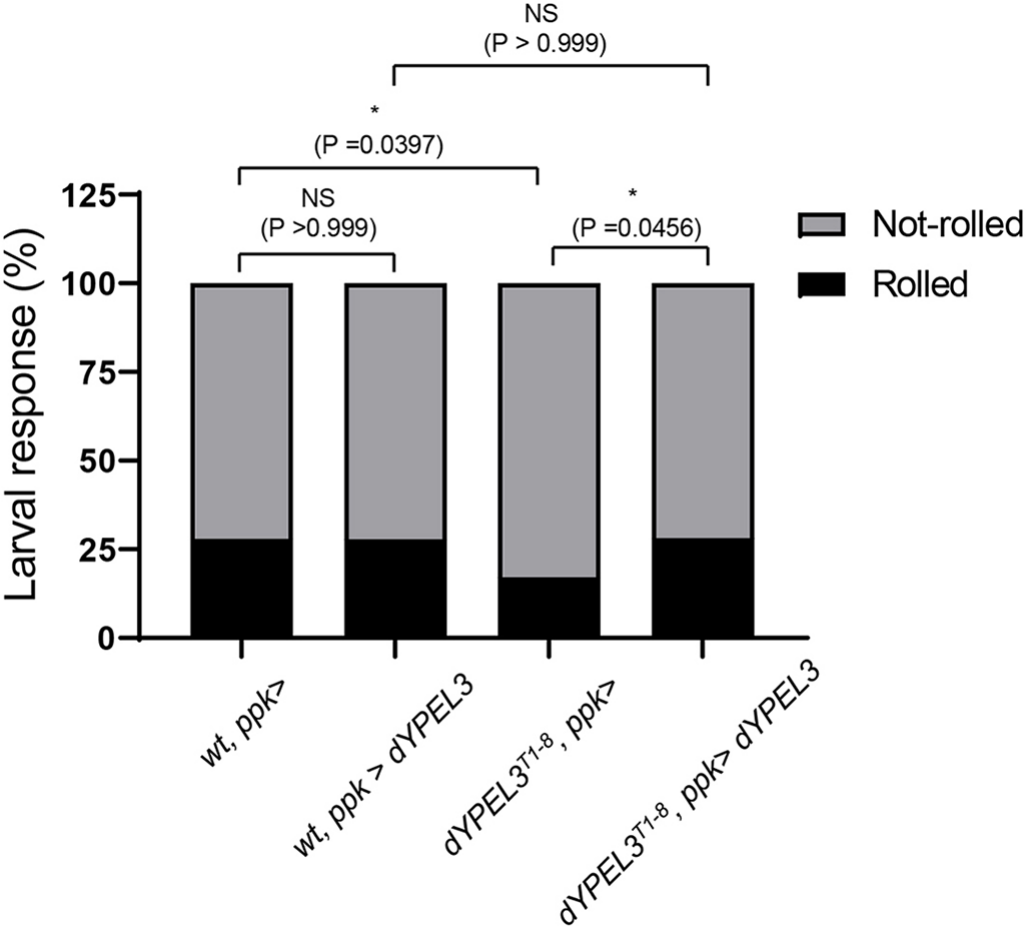
**The nociceptor-specific expression of dYPEL3 in *dYPEL3* frameshift mutant rescues AITC-mediated larva rolling behavior.** AITC-induced nociceptive behavior was measured. The nociceptor-specific expression of dYPEL3 was achieved using *ppk-GAL*. The number of larvae that exhibited complete rolling behavior was scored and expressed as a percentage (*n*=118 for *wt*, *ppk>*; *n*=115 for *wt*, *ppk>dYPEL3*; *n*=163 for *dYPEL3^T1-8^*, *ppk>*; *n*=103 for *dYPEL3^T1-8^*, *ppk>dYPEL3*). The Chi-squared test was performed between the groups. NS, non-significant; *P<0.05.

If *dYPEL3* frameshift mutation is loss of function, the defects in these mutants may be rescued by the expression of wild-type dYPEL3. The nociceptors, but not Basin-4 neurons, express dYPEL3 (Fig. 3A; Fig. S4). Thus, we expressed dYPEL3 specifically in nociceptors using *ppk-GAL4* (Grueber et al., 2007) in wild-type and *dYPEL3^T1-8^* larvae and tested AITC-induced nociceptive behavior. We found that expressing dYPEL3 in *dYPEL3^T1-8^* completely rescued defective rolling behavior induced by AITC (Fig. 8), whereas expressing dYPEL3 in wild type had no effect.

Taken together, these results strongly support a model that the frameshift in human *YPEL3* causes *YPEL3* loss of function, and that YPEL3 acts in presynaptic neurons to positively regulate synaptic contact.

## DISCUSSION

The biological functions of the YPEL gene family, including *YPEL3*, are poorly understood. Moreover, whether the identified *YPEL3* frameshift mutation is pathogenic is unknown. *Drosophila* provides a powerful tool for analyzing disease-relevant human gene mutations (Bellen et al., 2019). In this study, we report a *Drosophila* model of human *YPEL3* mutation and demonstrate that the disease-relevant *YPEL3* frameshift mutations are pathogenic in the nervous system.

The YPEL gene family is highly conserved across eukaryotes ranging from yeast to human. Likewise, our homology analysis indicated a strikingly high homology in gene sequences between human and *Drosophila YPEL3* (80% identity, Fig. 1B). Interestingly, it appears that the sequence homology extends even to the nucleotide level since the analogous frameshift mutation gave rise to the generation of similar amino acid sequences in the ectopic sequences in *dYPEL3^T1-6^* (Fig. 1C). Given such high sequence homology, we envision that the functions of human *YPEL3* and *Drosophila YPEL3* are also conserved. The YPEL family can be subdivided into two categories. Human YPEL1, YPEL2, YPEL3 and YPEL4 belong to one with high homology with each other, while YPLE5 constitutes a distinct family (Hosono et al., 2004). In *Drosophila*, there is only a single homolog of human YPEL1-YELP4, CG15309 (Fig. 1B). Because the tissue expression patterns of YPEL genes are complex in human and mice (Hosono et al., 2004), the single YPEL gene makes *Drosophila* advantageous as a model for studying YPEL3-induced pathogenesis.

In human and mice, *YPEL3* is ubiquitously expressed, as based on results from RT-PCR experiments (Hosono et al., 2004). Northern blot analysis of murine tissues shows relative enrichment of YPEL3 in brain and liver tissue (Baker, 2003). Our results based on a gene-trap *Drosophila* line indicates that dYPEL3 is expressed in subsets of neurons, but not in glia (Fig. 2B,C). The human patient exhibited multiple neurological symptoms in the PNS, but had normal cognition (the NIH-Undiagnosed Diseases Program). Interestingly, dYPEL3-GAL4 was selectively expressed in nociceptors and mechanosensors in the PNS (Fig. 3A; Fig. S2). Furthermore, *YPEL3* frameshift mutations reduced nociceptive behavior (Fig. 3B). These results suggest that at least some of the neurological symptoms in the human patient originate from neurons that express YPEL3.

How does the *YPEL3* frameshift mutation cause sensory deficits? The gross neuronal development of nociceptors was not altered by the *dYPEL3* mutations (Fig. 4). Calcium-imaging experiments showed that activation of nociceptors by AITC was not altered in *dYPEL3^T1-8^* mutants (Fig. 5B). Rather, *dYPEL3^T1-8^* reduced Basin-4 responses to nociceptor stimulation (Fig. 5A). This suggests that the neurotransmission from nociceptors to their postsynaptic neurons is reduced by the *dYPEL3* frameshift mutation. This conclusion is corroborated by the finding that the syb-GRASP signal between nociceptors and Basin-4 was reduced (Fig. 6). Since the syb-GRASP technique requires synaptic release (Macpherson et al., 2015), it is possible that the decrease in syb-GRASP signals reflects reduced activity or synaptic release in nociceptors in the mutants. Alternatively, the reduction in syb-GRASP signals might be due to a reduced number of synapses in the mutants. Additional techniques are needed to discern these possibilities. Nevertheless, the results from calcium imaging and syb-GRASP experiments consistently show reduced synaptic transmission from nociceptors to the SON Basin-4. Because Basin-4 activation is central to nociceptive behavior (Ohyama et al., 2015), the reduced synaptic transmission from nociceptors to Basin-4 is likely responsible for the reduction in nociceptive behavior in *dYPEL3* mutants. It is intriguing that the human patient has peripheral symptoms of hypotonia and areflexia; both may arise from reduced synaptic transmission.

We observed that AITC-elicited GCaMP signals persisted over a few minutes in both nociceptors and Basin-4 neurons (Figs 5A,B and 7C). Since AITC does not induce continuous larva rolling over such a long period, this implies the presence of an acute adaptation to AITC stimulation in the nociceptive circuit. It is possible that the *ex vivo* GCaMP measurement does not fully recapitulate neural activity *in vivo*. Nevertheless, our results indicate that the *dYPEL3* mutations significantly reduce calcium increase in Basin-4 neurons.

How does the *YPEL3* frameshift mutation affect *YPEL3* gene function? Our results suggest that *YPEL3* frameshift mutations cause loss of function. The behavioral, circuit and synaptic phenotypes were almost identical between *dYPEL3* frameshift (*dYPEL3^T1-8^*) and knockout (*dYPEL3^KO^*) mutants (Figs 3, 5, 6 and 7). Since nociceptors, but not Basin-4, express *dYPEL3* (Fig. 3A; Fig. S4), *dYPEL3* mutations likely affect presynaptic functions. Consistently, the behavioral phenotype in *dYPEL3^T1-8^* was completely rescued by expressing wild-type dYPEL3 in nociceptors (Fig. 8). Taken together, these findings suggest that YPEL3 functions in presynaptic neurons to regulate synaptic transmission, and that frameshift mutations in *YPEL3* result in loss of *YPEL3*.

The molecular function of YPEL3 is unclear. It contains predicted zinc-finger motifs (Hosono et al., 2004). The zinc-finger motifs in a YPEL domain of the yeast protein Mis18 is important for the folding of the YPEL domain, which mediates the centromeric localization of Mis18 (Subramanian et al., 2016). The YPEL domain in Mis18 has ∼20% sequence similarity to YPEL proteins. Since zinc-finger motifs are common in regulators of gene expression, we suspect that the frameshift mutation of YPEL3 may change gene expression. Indeed, overexpression of YPEL3 increased the expression of GSK3β to suppress the epithelial-mesenchymal transition (Zhang et al., 2016). It is interesting to note that GSK3β has been implicated in synaptogenesis (Cuesto et al., 2015). Thus, it will be important to determine whether YPEL3 regulates the expression of genes involved in synapse formation and maintenance and investigate how *YPEL3* frameshift mutations affect this process.

Overall, we generated a *Drosophila* model of the human *YPEL3* frameshift mutation and found that the *YPEL3* variant leads to deficits in synaptic transmission. We further demonstrate that the frameshift mutation causes loss of *YPEL3* function. In addition, this study establishes YPEL3 as a regulator of synaptogenesis or maintenance. Future studies that identify the molecular mechanisms underlying the function of YPEL3 will provide insights into therapeutic treatments of disorders caused by *YPEL3* mutations.

## MATERIALS AND METHODS

### *Drosophila melanogaster* strains

*Drosophila* strains were kept under standard condition at 25°C in a humidified chamber. The following strains were used: *w^1118^* (3605), *ppk-GAL4* (Grueber et al., 2007), *ppk-LexA* (Gou et al., 2014), *UAS-syb::spGFP1-10* (Macpherson et al., 2015), *LexAop-CD4::spGFP11* (Macpherson et al., 2015), *UAS-FRT-rCD2-stop-FRT-CD8::GFP* (Wong et al., 2002), *hs-FLP* (Nern et al., 2011) (55814), *UAS-CD4-GFP* (35836), *UAS-GCaMP6f* (Mutlu et al., 2012) (42747), *LexAop-GCaMP6f* (Mutlu et al., 2012) (44277), *CG15309-GAL4* (Gohl et al., 2011) (62791), *nos-Cas9* (Port et al., 2014) (54591), *GMR57F07-GAL4* (Jenett et al., 2012) (46389), *GMR57F07-lexA* (Pfeiffer et al., 2010) (54899), *UAS-GtACR1* (Mohammad et al., 2017), *NompC-lexA* (Shearin et al., 2013) (52241) and a Cre-recombinase expressing fly line (1501, RRID:BDSC_1501). The numbers in parentheses indicate the stock numbers from the Bloomington *Drosophila* Stock Center.

### The generation of *dYPEL*3 mutants

The CRISPR/CAS9-mediated in-del mutation was used to generate *dYPEL3* frameshift mutant flies. A guide RNA construct was generated in pCFD:U6:3 (Port et al., 2014) with guide RNA sequences that target the middle of the *dYPEL3* coding exon. The standard transformation procedure was performed to generate a transgenic line. The transformants were crossed with *nos-Cas9* (Port et al., 2014) flies to induce in-del mutations in germ cells. The resulting progeny were screened for the desired mutations by the genomic PCR of *CG15309* following the Sanger sequencing. The genetically null *dYPEL3* allele (*dYPEL3^KO^*) was generated using CRISPR/Cas9-mediated homology directed recombination (HDR). The HDR donor construct was built using pBluescript as a backbone, which includes *3XP3:RFP* (for expressing DsRed in eyes) that is flanked by loxP sequences (Lin and Potter, 2016) and two homology arms (∼700 bp and ∼1 kb for right and left arms, respectively). The homology arms were amplified from *w^1118^* flies. Two guide RNA constructs that target near the start and the end of *dYPEL3* open reading frame (ORF) were cloned in pCFD:U6:3 (Port et al., 2014). The two guide RNA constructs and the HDR donor construct were co-injected into *w^1118^*, *nos-Cas9* fly embryos. Flies were screened for eye expression of DsRed, and successful integration of the donor construct was confirmed by a PCR-based genotyping. The eye-specific DsRed cassette was removed by crossing the flies carrying the donor construct and those expressing Cre recombinase. Generation of *UAS-dYPEL3* was done using pUASTattB plasmid and *dYPEL3* ORF that was amplified from *w^1118^* genomic DNA. Standard methodology was used to generate transformants (Bischof et al., 2007).

### AITC-induced nociceptive behavior

AITC (Sigma-Aldrich) was prepared in DMSO, dissolved in water to a final 25 mM concentration, and incubated on a rocker for 3 days before use. Fly embryos were grown for 5 days in a 12 h light/dark cycle at 25°C in a humidified incubator. The third-instar larvae were moved to room temperature for 1 h, gently scooped out of food, washed in tap water and placed on a grape-agar 24-well plate that had been covered with 300 µl AITC solution (25 mM). Their behavior was recorded with a digital camera for 2 min and the number of larvae showing complete rolling behavior (minimum 360° rolling) was manually analyzed (Honjo et al., 2012). The experiments were paired for the wild-type control (*w^1118^*) and *dYPEL3* homozygous mutant larvae. Experiments were repeated three times on different days with different AITC preparations. All three trials were combined for statistical analysis.

### Calcium imaging

Live calcium imaging was performed using GCaMP6f (Mutlu et al., 2012). Briefly, wandering third-instar larvae – wild-type control males or *dYPEL3^T1-8^* hemizygotes – were dissected in a modified hemolymph-like 3 (HL3) saline (Stewart et al., 1994) (70 mM NaCl, 5 mM KCl, 0.5 mM CaCl_2_, 20 mM MgCl_2_, 5 mM trehalose, 115 mM sucrose and 5 mM HEPES, pH 7.2). Glutamate (10 mM) was added to the HL3 solution to prevent muscle contractions and sensory feedback. The GCaMP signal was recorded in the entire volume of nociceptor axon terminals or Basin-4 cell bodies. Live imaging was performed with a Leica SP5 confocal system or a custom-built spinning disk microscope equipped with an extra-long-working distance 25× water objective with 2-µm step sizes. The membrane tdTomato proteins were expressed along with GCaMP6f and used as an internal normalization control for both lateral and focus drifting. The basal GCaMP signal was recorded for a duration of 30 s to generate baseline fluorescence (*F*_0_), and then the samples were treated with AITC (10 mM) in HL3 while being continuously recorded for an additional 150 s. The 3D time-lapse images were collapsed to 2D time-lapse images by using the maximum Z-projection in ImageJ software (NIH). The region of interest was selected in the axonal projection of nociceptors or in the cell bodies of Basin-4. The ImageJ Time Series Analyzer plugin was used to quantify the fluorescence intensity of GCaMP6f. The cumulative GCaMP was calculated from the GCaMP tracing from AITC treatment (t=30 s) to the end of recording (t=180 s).

### Immunostaining

Immunostaining was performed essentially as previously reported (Kim et al., 2013). The primary antibodies used were as follows: chicken anti-GFP (AB_2307313, Aves Laboratories; 1:2500), rabbit anti-RFP (600-401-379-RTU, Rockland Immunochemicals; 1:5000), rat anti-Elav (9F8A9, Developmental Studies Hybridoma Bank; 1:100) and mouse anti-Repo (8D12, Developmental Studies Hybridoma Bank; 1:5). The secondary antibodies were from Jackson ImmunoResearch and used at 1:500 dilution: Cy2- or Cy5-conjugated goat anti-chicken, Cy2- or Cy5-conjugated goat anti-mouse, Cy5-conjugated goat anti-rabbit and Cy3-conjugated goat anti-rat. Confocal imaging was performed with a Leica SP8 confocal system or a custom-built spinning disk confocal microscope equipped with a 63× oil-immersion objective with 0.3-µm step size. The resulting 3D images were projected into 2D images using a maximum projection method.

In order to report the relative synaptic contact between the nociceptors and their postsynaptic partners, syb-GRASP was performed in the male larvae from a wild-type control (*w^1118^*), *dYPEL3^T1-8^* or *dYPEL3^KO^* hemizygotes. Syb::split-GFP1-10 (Macpherson et al., 2015) was expressed in nociceptors. CD4::split-GFP11 (Macpherson et al., 2015) was expressed in Basin-4 neurons. The polyclonal chicken anti-GFP antibody (Aves Laboratories) recognizes the split-GFP1-10 and the reconstituted GFP protein, while the mouse anti-GFP antibody (G6539, Sigma-Aldrich) recognizes only the reconstituted GFP. Therefore, the mouse anti-GFP antibody was used to measure the GRASP signal (anti-GRASP; 1:100) and the polyclonal chicken anti-GFP antibody was used as an internal control for normalizing the GRASP signal. The fluorescence images were acquired to minimum signal saturation for quantitation. The mean fluorescence intensities of anti-GRASP and anti-split-GFP1-10 from each hemi-neuropil segment (segments 4, 5 and 6) were measured from the confocal images.

### Assessment of dendrite development in nociceptors

The membrane GFP, mCD8::GFP, was specifically expressed in nociceptors using *ppk-GAL4* in a wild-type control (*wt*) and *dYPEL3* frameshift mutants (*dYPEL3^T1-8^*). Total length of dendrites was measured from the male larvae of *wt* and *dYPEL3^T1-8^* using the Simple neurite tracer plugin (Longair et al., 2011) in ImageJ software. Sholl analysis was conducted using the Sholl analysis plugin in ImageJ software (Ferreira et al., 2014).

### Analysis of presynaptic arbors of single nociceptors

The flip-out (Wong et al., 2002) experiment was performed to visualize the terminal axon arbors of single nociceptors. A flip-out cassette (*FRT-rCD2-stop-FRT-CD8::GFP*) and a heat-shock inducible Flippase (FLP) was introduced either in a wild-type control (*w^1118^*) or in *dYPEL3^T1-8^* mutants along with *ppk-GAL4*. The 3-day-old larvae grown in grape-agar plate were heat shocked for 15 min in a 37°C water bath and allowed one more day of growth at 25°C before being dissected and processed for immunostaining and imaging. The total presynaptic arbor length was manually measured using ImageJ software. Branches shorter than 5 µm were excluded from the analysis.

### Experimental design and statistical analysis

All statistical analysis was performed two-tailed using Prism version 7.04 (GraphPad Software). The Chi-square with Fisher's exact test was used for nociceptive rolling behavior. The Mann–Whitney test was used for calcium imaging (GCaMP) and GRASP experiments. Unpaired Student's test was used for presynaptic arbor size and dendritic development analysis. Two-way ANOVA was used for Sholl analysis. *P*<0.05 was considered statistically significant.

## Supplementary Material

Supplementary information
