# Supplementary material for: Frameshift mutations of YPEL3 alter the sensory circuit function in Drosophila
Source: Dis Model Mech. 2020 Jun 3;13(6):dmm042390. doi: 10.1242/dmm.042390 (PMC7286299; doi:10.1242/dmm.042390)
Supplement: Supplementary information [file dmm-13-042390-s1.pdf]

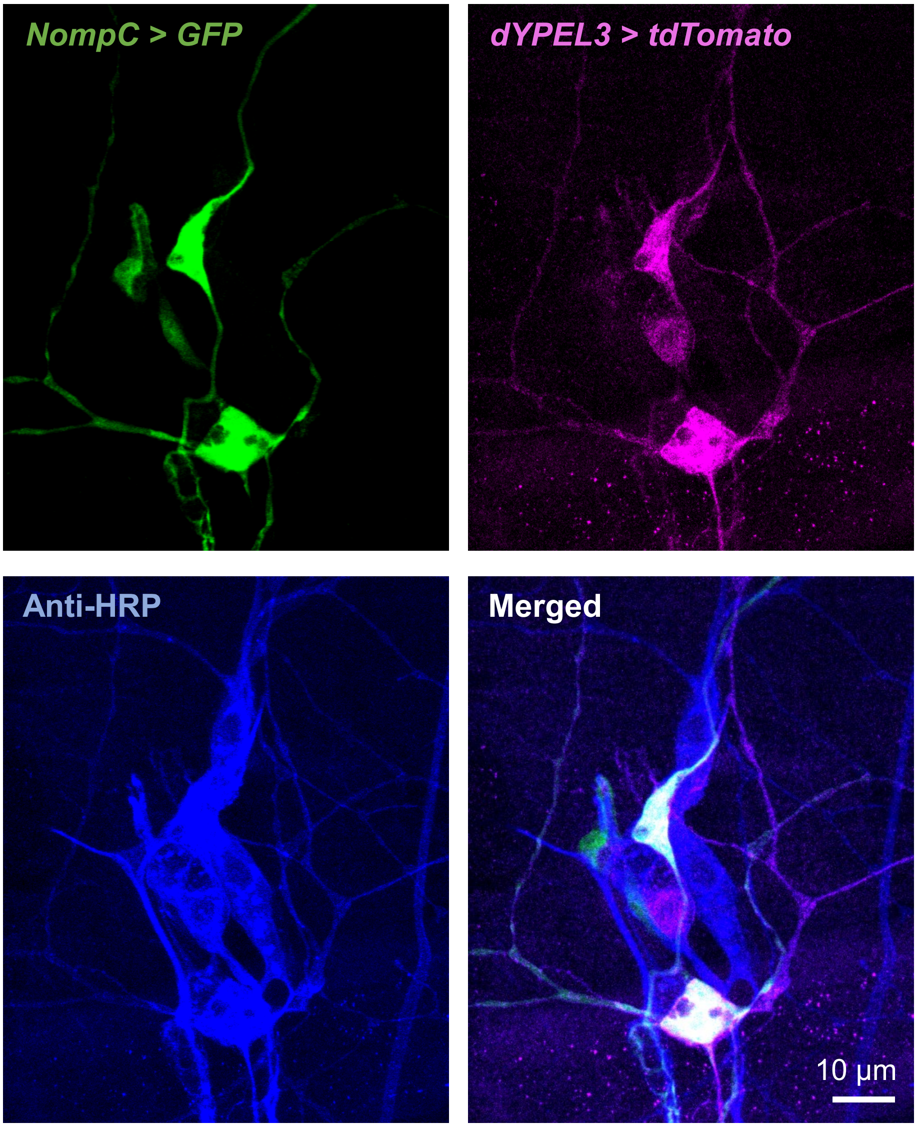

Figure S1. Class III da mechanosensory neurons express *dYPEL3*
GFP was expressed under a mechanosensory neuron marker, NompC-LexA. Membrane targeted tdTomato was expressed under *dYPEL3-GAL4* (magenta, anti-RFP). The dorsal cluster of the larval da neurons were imaged in wandering 3rd instar larvae Note that GFP-positive class III da neuron express tdTomato.

Disease Models & Mechanisms • Supplementary information

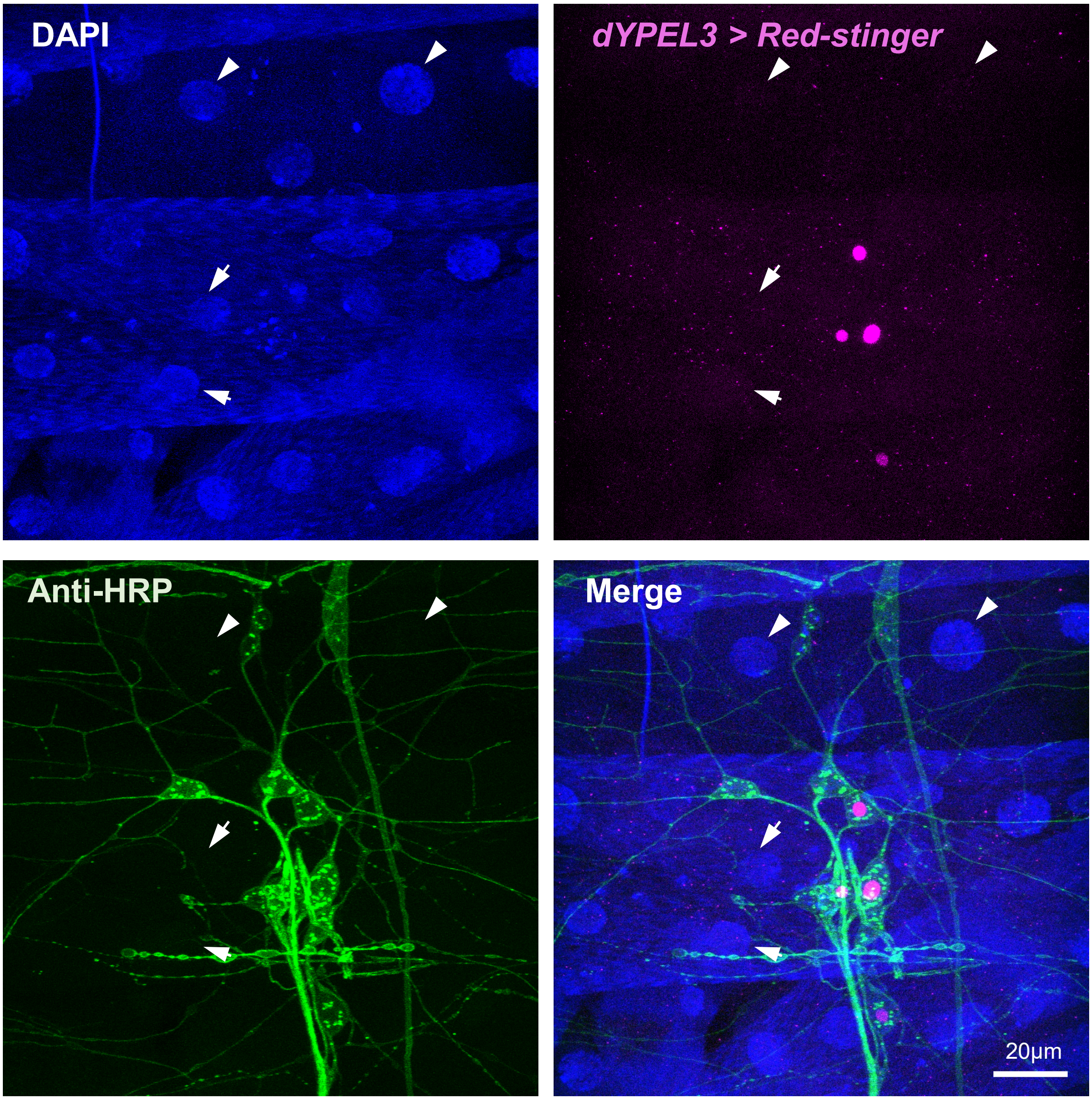

Figure S2. Muscle and epidermal tissue do not express *dYPEL3*.
Red-stinger, a nucleus-targeted red fluorescence protein was expressed under *dYPEL3-GAL4* (magenta, anti-RFP). Wandering 3rd instar larvae were dissected to reveal their body wall that contains both larval muscles and epidermis along with the PNS neurons (green, anti-HRP stain). Cell nuclei were labeled with DAPI (blue) to identify muscle and epidermal cells. Note that the nuclei from both muscle and epidermal cells are devoid of RFP signal.

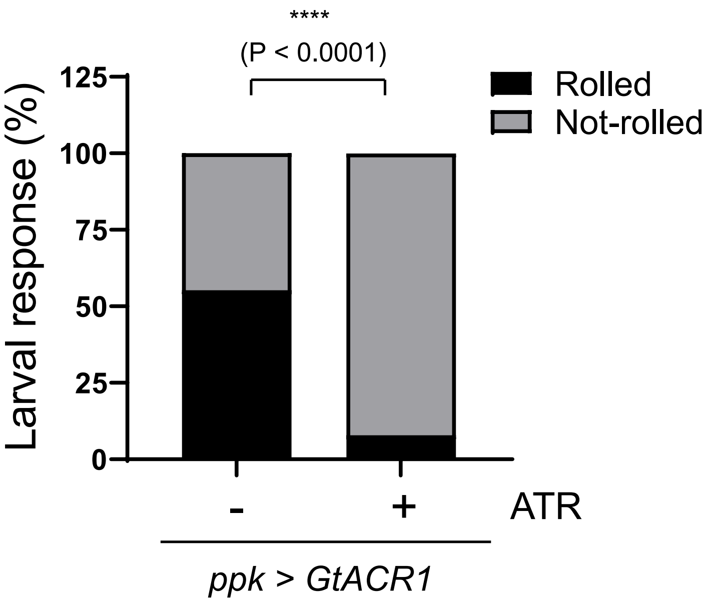

Figure S3. Nociceptors are essential for AITC-induced larva rolling behavior. Light-sensitive *Guillardia theta* anion channelrhodopsin-1 (GtACR1) was expressed in the larval nociceptors (C4da neurons) using *ppk-GAL4*. The larvae were grown the presence and the absence of all-trans-retinal (ATR). AITC-induced rolling behavior was measured under 60 μWatt/mm^2 of 515 nm LED illumination. The activation of GtACR1, hence the suppression of the larval nociceptors dramatically reduced AITC-induced larva rolling behavior.

Disease Models & Mechanisms • Supplementary information

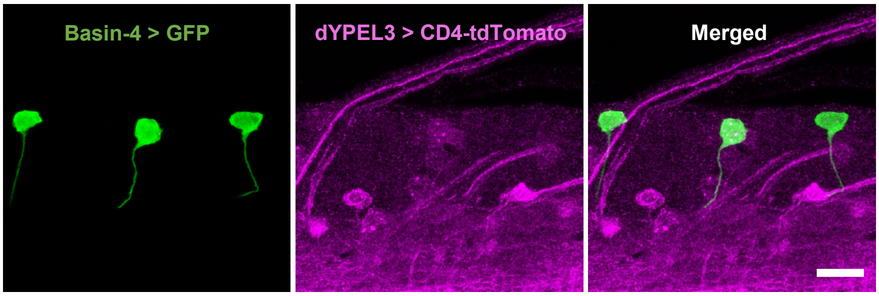

Figure S4. Basin-4 neurons do not express *dYPE3*.
A membrane red fluorescent protein, CD4-tdTomato, was expressed under *dYPEL3-GAL4* (magenta). GFP was expressed under Basin-4 specific LexA (green). Note that the cells expressing GFP does not overlap with the cells that express *dYPEL3-GAL4*. Scale bar = 10 μm

Disease Models & Mechanisms • Supplementary information
